# Would you be impressed: applying principles of magic to chatbot conversations

**DOI:** 10.3389/frobt.2024.1256937

**Published:** 2024-04-24

**Authors:** Sarah Rose Siskind, Eric Nichols, Randy Gomez

**Affiliations:** ^1^ Hello SciCom, New York, NY, United States; ^2^ Honda Research Institute Japan, Co, Ltd, Saitama, Japan

**Keywords:** magic principles, chatbot, conversations, dialogue systems, social robots

## Abstract

A magician’s trick and a chatbot conversation have something in common: most of their audiences do not know how they work. Both are also constrained by their own limitations: magicians by the constraints of biology and physics, and dialogue systems by the status of current technology. Magicians and chatbot creators also share a goal: they want to engage their audience. But magicians, unlike the designers of dialogue systems, have centuries of practice in gracefully skirting limitations in order to engage their audience and enhance a sense of awe. In this paper, we look at these practices and identify several key principles of magic and psychology to apply to conversations between chatbots and humans. We formulate a model of communication centered on controlling the user’s attention, expectations, decisions, and memory based on examples from the history of magic. We apply these magic principles to real-world conversations between humans and a social robot and evaluate their effectiveness in a Magical conversation setting compared to a Control conversation that does not incorporate magic principles. We find that human evaluators preferred interactions that incorporated magical principles over interactions that did not. In particular, magical interactions increased 1) the personalization of experience, 2) user engagement, and 3) character likability. Firstly, the magical experience was “personalized.” According to survey results, the magical conversation demonstrated a statistically significant increase in *“emotional connection”* and *“robot familiarity.”* Therefore, the personalization of the experience leads to higher levels of perceived impressiveness and emotional connection. Secondly, in the Magical conversation, we find that the human interlocutor is perceived to have statistically-significantly higher engagement levels in *four* of *seven* characteristics. Thirdly, participants judged the robot in the magical conversation to have a significantly greater degree of *“energeticness*,*”“humorousness,”* and *“interestingness*.*”* Finally, evaluation of the conversations with questions intended to measure contribution of the magical principals showed statistically-significant differences for *five* out of nine *principles*, indicating a positive contribution of the magical principles to the perceived conversation experience. Overall, our evaluation demonstrates that the psychological principles underlying a magician’s showmanship can be applied to the design of conversational systems to achieve more personalized, engaging, and fun interactions.

## 1 Introduction

“Any sufficiently advanced technology is indistinguishable from magic.” —Arthur C. Clarke (1973, p.21).

The history of magic and robotics are entwined. The Mechanical Turk, an unbeatable automaton chess player, amazed eighteenth-century audiences with feats that seemed far beyond what machines were thought capable of at the time (Standage, 2002). In fact, it was beyond what a machine was capable of at the time. The Mechanical Turk was a magician’s trick. But its legacy was enduring in the popular imagination and now, after IBM’s Deep Blue supercomputer, a chess master machine is real. Magic can become reality.

Conversations with inanimate conversational agents require a kind of suspension of disbelief rather than outright gullibility. Unlike interacting with most computer programs, users interact with a chatbot as if it is a fellow human. Designers intend this anthropomorphism; for example, some systems only respond when called by their name, and many systems do not respond to profanity (Adam et al., 2021). Most (54%) of smart speaker owners report that they say “please” when talking to their device (Pew Research Center, 2019). This is a kind of voluntary magical thinking much like the state of mind of the audience in a magic show. In this sense, meaningful conversations with dialogue systems are less like deception and a bit more like fantasy.

Magicians and conversational systems alike try to present a “special” experience. In the case of the magician, they often pick a volunteer to introduce into the performance an element of randomness that makes the interaction unique. Audiences do not appreciate a performance that appears staid and formulaic. The “trick” is that magicians are highly formulaic performers. Similarly, chatbots should be discreet about their formulaic nature in order to create personalized interactions.

It is not just the performance that should be unique, but the performer too. Magicians usually infuse their acts with their character. A trick by Penn and Teller, for example, is often hilarious and informal, quite different from the gravitas of David Copperfield (Trillin, 1989; Gaydos, 2013). Conversational agents, too, may benefit from infusing their interactions with personality. Many notable conversational agents today are chatbots where the user is expected to prompt the bot with a query and receive a response from a generic and neutral character. But a conversational agent that initiates and guides a user through an interaction may, if given a distinct character, be perceived as far more memorable, likable, and awe-inspiring (Nichols et al., 2022a).

In this paper, we explore the design of chatbot conversation strategies influenced by the communication techniques of magicians. To be clear, these interactions were inspired by the psychological principles underlying a magician’s showmanship, but the principles are broader than just tricks or illusions. They influence a larger communication style that incorporates moments of suspense, awe, and humor, in essence, showmanship. With this in mind, we identify ten key magic principles, give examples of their application in the world of magic, and then show how they could be applied to conversations. Then, targeting Haru the social robot, we create a proof-of-concept conversation implementation that includes an example from each magic principle. Finally, we confirm the contributions of these magic principles to chatbot conversations with humans through an elicitation study with human evaluators. With the goal of creating a more engaging conversation, we tested the users’ perceived levels of personalization, engagement, and character likability.

The rest of this paper is organized as follows: in Section 2, we introduce our target social robot and dialogue framework; in Section 3, we introduce the magic principles that inform our chatbot conversation strategy; in Section 4, we present an elicitation survey that evaluates our approach; in Section 5, we discuss the findings of our survey; in Section 6, we outline relevant work; and, finally, in Section 7, we conclude.

## 2 Related research

Much research has been conducted on conversation with robots. Mavridis. (2015) summarize recent work, covering verbal and non-verbal aspects of communication, as well as desiderata for interactions. Our focus on likability is similar to their *affective communication* concept. Zhang et al. (2016) conduct a research review, focusing on 1) *affective systems with dialog*, 2) *task-driven memory with dialog*, and 3) *chat-driven memory with dialog*. Liu and Zhang (2019) focus on linguistic issues in human-robot cooperation and discuss challenges and approaches to understanding, planning, and executing instructions in natural language. Personalization based on previous interactions between a robot and a user has been explored as a means of developing rapport has been shown in prior studies to improve users’ perceptions and attitudes toward robots (Lee et al., 2012).

Recently, research has been conducted involving humanoid robot magic shows, where a robot acts not as a magician’s prop but as the magician itself. Jeong et al. (2022) use a robot magic show to test a new method for analyzing interactions in performance art with a view towards clarify the artist’s intent. Yang et al. (2023) describe a humanoid robot performing pen tricks while trying on different personalities. Both studies focus on humanoid robots, and not robots and chatbots in general.

## 3 Target robot and dialogue framework

**FIGURE 1:**
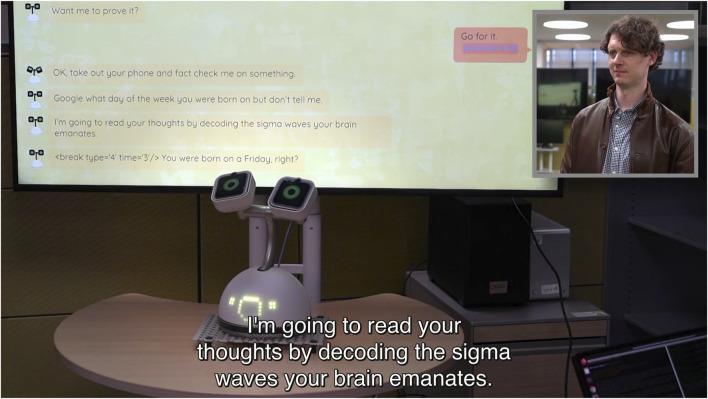
Aspiring mentalist Haru the robot interacts with a human volunteer.

As our target robot, we selected the social robot Haru (Gomez et al., 2018; Gomez et al., 2020a; see Figure 1). Haru is an experimental tabletop robot for multimodal communication that uses verbal and non-verbal channels for interactions. Haru’s design is centered on its potential to communicate emotions through richness in expressivity (Gomez et al., 2020b).

Haru has five motion degrees of freedom, namely, *base rotation*, *neck leaning*, *eye stroke*, *eye rotation* and *eyes tilt*, which allow it to perform expressive motions. Furthermore, each of the eyes includes a three-inch TFT screen display in which the robot eyes are displayed. Inside the body, there is an addressable LED matrix (the mouth). Haru can communicate via text-to-speech (TTS), through animated routines, projected screen, and more. Haru’s range of communicative strategies positions the robot as a potent embodied communication agent capable of long-term interaction with people. Haru has been deployed in a variety of scenarios, including classroom companion, interactive storyteller (Nichols et al., 2021a), and, of course, conversational partner (Nichols et al., 2022a).

Haru’s TTS voice (Nichols et al., 2021c) was designed to convey a wide range of emotions and achieves this by combining several *vocal genres*—special variants built on a base TTS voice that encapsulate a specific delivery style—to form an expressive but consistent voice. Haru’s vocal genres include cheeky, empathetic, high-energy, question, sad, serious, whiny, and whisper-yell. The vocal genres were designed to maximize emotive coverage by being flexible: e.g., serious can express anger, urgency, or worry; cheeky can express playfulness or sarcasm; and whiny can express anger, disgust, or fear.

**FIGURE 2:**
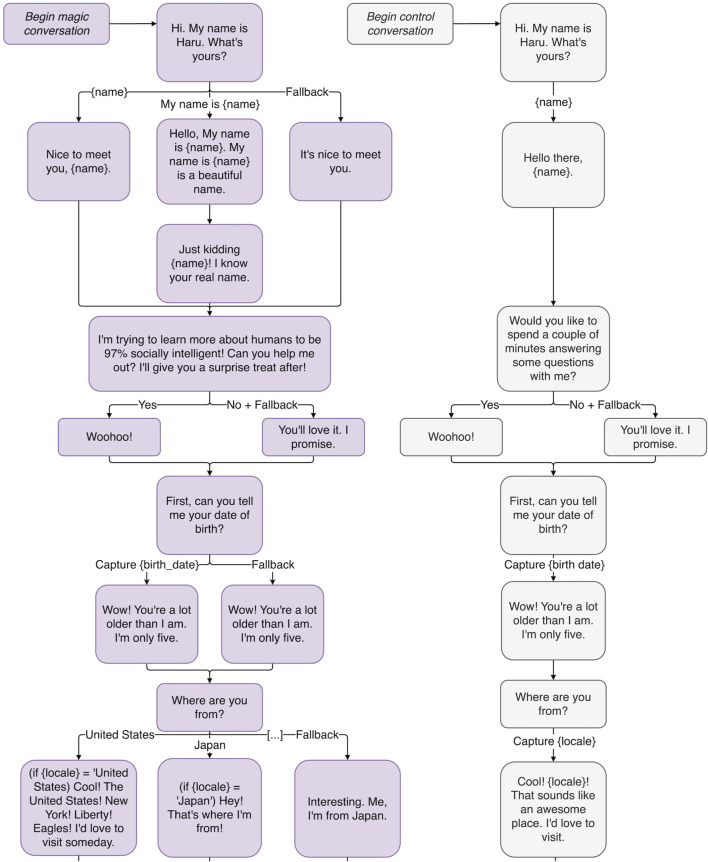
The beginning of a conversation tree implementing a proof-of-concept magic principles-inspired conversation, illustrating the magic principles: *Have a Story, Misdirection*, *Sucker Gag, and Playing the Odds.*

Haru is equipped with a custom dialogue framework (Nichols et al., 2022a) that incorporates a conversational memory with hierarchical, topic-module-based conversation organization and navigation, allowing for the development and deployment of curated conversational content that is both dynamic and personalized. Memory variables, such as {name} or {sport} (see Figures 2, 4, 5 for more examples), let conversation creators specify information for Haru to learn from users that can be recalled and referred to later in the conversation. Its tree-based navigation structure allows for the definition of fallback paths to elegantly handle unexpected responses from users. This dialogue framework has been applied in a variety of scenarios ranging from small talk, dialogue-based gameplay, group interactions in the classroom, and conversations with hospital patients.

Haru’s expressive capabilities, unique personality, and flexible dialogue framework make it an ideal platform for exploring the application of magic principles to chatbot conversation design.

## 4 Magic principles

Magicians are an ancient profession, with magic techniques traditionally being handed down orally or through texts that are not readily accessible to non-practitioners. Categorizing magic tricks according to their methods and intended effects also has a controversial history. For instance, in Kuhn et al., 2014, Kuhn et al., 2014 a novel taxonomy of misdirection is directly compared to at least four earlier taxonomies that the authors find wanting. Lamont (2015) argues that the enterprise may be doomed to fail from the start, concluding that even though “it is certainly possible to construct another inventory of magic tricks, and to describe a variety of relationships between effects and methods […] it is difficult to see how a complete list of magic tricks could be compiled, or why any list might be “more natural” than another.” The magic principles that follow are by no means exhaustive or universally accepted. They were derived by us from informal interviews with working magicians, and even our interview subjects gave conflicting answers and disagreed on terminology. Additionally, only those that seemed particularly relevant to chatbot writers were included. These are not even, necessarily, the most important principles to practicing magicians. Sleight-of-hand involving everyday objects, for instance, is closely associated with close-up magic (Lamont and Wiseman, 1999, p.110), but its usefulness to designing dialogue systems is less obvious. Similarly, while forcing techniques involving visual saliency may be of great interest to game designers (Kumari et al., 2018), chatbot designers would likely do better to focus just on those techniques that involve words.

We look at ten major principles. Magicians have learned that giving a trick *“a story”* behind it can help it to feel motivated. The performer must carefully direct the attention of the audience towards the moments of awe and *“misdirect”* away from a potential disappointment. Teasing the audience with a *“sucker gag”* actually builds rapport. By *“playing the odds,”* a performer does not need to have a response to every possible contingency, just the most likely possibilities. If a normal trick can be given an *“emotional connection,”* it vastly improves the impressiveness of the trick. Repeating or even exaggerating the impressiveness of a trick can *“drive the point home.”* Reintroducing an element from the beginning of a performance at its conclusion can create a sense of completion and satisfaction, so the performer may remind the audience to *“remember the claim”* right before the final reveal. *“Forcing”* is a method by which the performer can control what happens in an interaction while giving the illusion that the audience has control. By *“having multiple outs,”* the performer can have multiple options for ways to end a trick depending on what works best. Finally, a magician can top off the entire interaction, after the audience thinks all the tricks have been revealed, with a final surprise or *“kicker.”*

### 4.1 Have a story

Magicians often do not just start silently pulling cards out of pockets or rabbits out of hats. Instead, they may motivate a trick or an entire performance by having a story about why they are doing what they are doing. The late, great magician Ricky Jay used the story of the Sword of Vengeance, taken from a samurai film, throughout the card trick he was performing (Illuminations Television, 1996). It added an element of gravitas. For comedic effect, Penn and Teller go on a rage-fueled tirade about TSA while performing tricks with a metal detector (Baskas, 2009). David Copperfield, for patriotic effect, famously made the Statue of Liberty disappear to show how easily our liberty can be lost (Cates Films, 1983).

A chatbot needs to have a compelling motive for talking to a human. Otherwise, conversations will feel stalled, like a hostage negotiator trying to get you to stay on the line while someone traces the call. In Figure 2, the reason Haru gives for starting a conversation with the user is that he is “trying to learn more about humans to be 97% socially intelligent!” His story is intended to add purpose and motivation to his conversations.

### 4.2 Use misdirection to ... hey look over there!

Magician Matthew Holtzclaw says, *“Magic does not happen in the hand, it happens in the audience’s mind.”* (2021, personal communication) Shifting the audience’s attention away from the magician’s actions, and making their routines appear magical, is the job of misdirection. Renowned magic book and blog The Jerz, (2017) cautions, *“Don’t think about misdirection as being about the direction of someone’s focus or interest. Instead, think about it as the direction of someone’s suspicion”*. Famed pickpocket Apollo Robbins uses a poker chip and some clever gags to distract a volunteer while he uses sleight of hand to steal his watch (TED, 2013). This is especially impressive because Robbins was in the middle of a talk about misdirection. Magician Harrison Greenbaum explains, *“Non-magicians often think that misdirection is moving an audience’s attention away from what we do not want them to see, but, in reality, it is about moving an audience’s attention towards something else.”* (2022, personal communication).

In their taxonomy of misdirection, Kuhn et al. (2014) divide misdirection techniques according to the psychological mechanisms they affect. Confusion—a sub-category of forgetting, itself a sub-category of memory misdirection—works by overloading the spectators memory, making it harder for them to remember all the details of the performance. Haru can use an extremely simplified version of this concept. For example, if Haru were to ask the user what their biggest fear is, maybe only four responses get their own dedicated conversation branches, with everything else going under fallback. To distract the user from Haru not having anything prepared for their specific phobia, and to make him look more informed than he actually is, Haru immediately shifts the subject: *“Scary! I wonder what that’s called. Do you know what a fear of confined spaces is called?”* This move reframes the conversation so that the fear is about technical phobia classification, and then he spins the conversation towards a question most people would feel the urge to answer (*claustrophobia*). Misdirection also works easily with Haru’s character because, as a childlike robot, he is easily distracted.

### 4.3 Give the audience trust issues with a “sucker gag”

**FIGURE 3:**
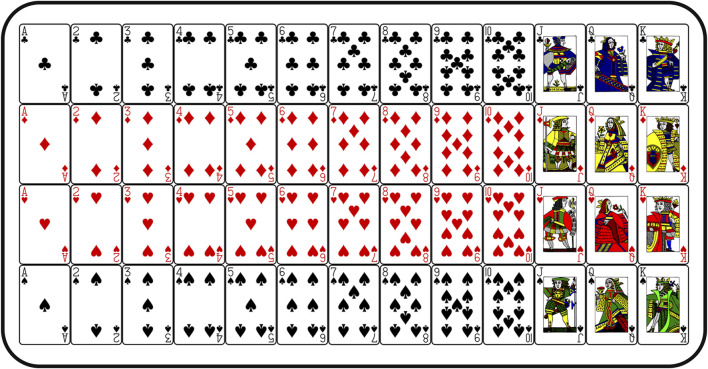
A 52-in-1 card.

Where misdirection is about playing with the audience’s attention, sucker gags play with the audience’s expectations. With these, the magician creates the expectation that something will happen—and then it does not. A classic example of a sucker gag is the 52-on-1 card. The magician predicts that the card they are holding is the same as the one the audience member will pick. The audience member picks a card, say the six of spades. Then the magician turns over their own card to reveal that it had all fifty-two possibilities printed on it (See Figure 3). The moment obviously contains no magic at all, and so is played for comedy. Sucker gags are to be distinguished from the similarly named term “sucker effect,” which describes situations in which the audience is misled into believing they know how a trick works only to have the rug pulled out from under them. It has been proven that implanting in a participant’s mind a false solution can prevent them from guessing the real one, even when the false solution is much more implausible, and even when the magician demonstrates it to be false (Thomas and Didierjean, 2016; Thomas et al., 2018).

In Figure 2, Haru fools the user when he pretends he thinks “My name is” is part of their name. This kind of gag or practical joke is in line with Haru’s prankster nature. Rarely would a real magician leave it at that, however. The sucker gag is generally just the first part of the trick, followed by an instance of actual magic. In “The Chicago Surprise”, Pop Haydn’s variation on “The Chicago Opener” or “Red Hot Mama”, the audience is made to believe that the magician has guessed the wrong card for one highly awkward moment before the volunteer looks at the card in her hand and realizes it has been replaced by the correct one (Haydn, 2014). Some escapist performers use the sucker gag to terrifying effect by making the audience think the trick has gone so wrong that the performer is in danger. For example, Demian Aditya tries to escape from a box filling up with sand, only to have the lid collapse and stagehands come out to hammer open the chamber (America’s Got Talent, 2017). Of course, one of those stagehands turns out to be Demian Aditya, the escapist, himself.

### 4.4 Playing the odds

How much do you relate to the following statement: *“Recently, you have had to recover from a disappointment?”* Odds are you can relate, since life is full of disappointments. The tendency of individuals to accept vague, general statements as uniquely applicable to themselves is known as the Barnum Effect. Barnum statements, and the related set of techniques known as “cold reading,” have been studied by researchers for many years (Forer, 1949; Hyman, 1981). These tactics are used most often by psychics and pickup artists because the “trick” relies partly on the gullibility of the participant and makes the participant feel understood. The late, amazing James Randi proved the effectiveness of “playing the odds” by debunking psychics and mystics of all kinds (WGBH-TV, 1993). He famously gave personalized horoscopes to a class of students and had them rate how accurate their reports were (apparently very accurate) only for the class to discover they had all been given the same horoscope.

A related phenomenon is the *Gray Elephants in Denmark* trick: most people when asked to think of an animal beginning with E will pick an elephant, when asked to think of that animal’s color will pick gray, and when asked to think of a country beginning with D will pick Denmark. Audiences are generally shocked when the mentalist says, *“That’s funny. I do not think there are gray elephants in Denmark.”* This is playing the odds, and as one of the few magic tricks capable of being done over the radio (Pogue, 1998, pp.263-265), it has lessons applicable to chatbot writing. Unlike Barnum statements, what the mentalist says is not vague at all. In fact, it is very specific. But note that, while the mentalist is forcing someone to pick an animal beginning with E (and a country beginning with D), no one is forcing them to pick an elephant (or Denmark). Just as someone is free to disagree with a Barnum statement, they are free here to say “Eagle.” The mentalist is simply aware, in both cases, of what the most likely answer will be. Similarly, when the user is offered a non-binary choice, instead of writing dialogue for every possibility, dialogue is written only for the most likely answers. For example, when Haru asks the user, *“So what’s your home country?”* (Figure 2) there is a high probability that the user will be from China, Japan, India or the US. If it is the last one, Haru is programmed with the response, *“America! New York! Liberty! Eagles! I’d love to visit someday.”* The user might assume that Haru has a response for every country in the world when, in reality, if the user is not from one of four countries, Haru will just shift the conversation to a different topic: *“Interesting. Me, I’m from Japan. What kind of sports do you watch?”* The user hopefully overestimates Haru’s abilities, which is intended to increase their engagement.

### 4.5 Establishing an emotional connection

According to Holtzclaw, *“Trust can be built with an audience member by adding an emotional component.”* (2021, personal communication) Childhood memories are very powerful in this respect. Early childhood memories are more likely than not to elicit emotional reactions (Kihlstrom and Harackiewicz, 1982; Howes et al., 1993). It is one thing for a magician to guess your card, but it is a much bigger psychological payoff if the magician guesses the name of the street you grew up on.

The entirety of mentalist Derek Delgaudio’s award-winning performance, “In and Of Itself,” employs this principle (Hulu Originals, 2021). In the culminating act, he guesses deeply personal identifying cards from each audience member. He may be employing simple memory techniques to memorize so many cards, or he may have a hidden tool to remember them all, but it is the personal nature of these cards that makes the “trick” so moving.

**FIGURE 4:**
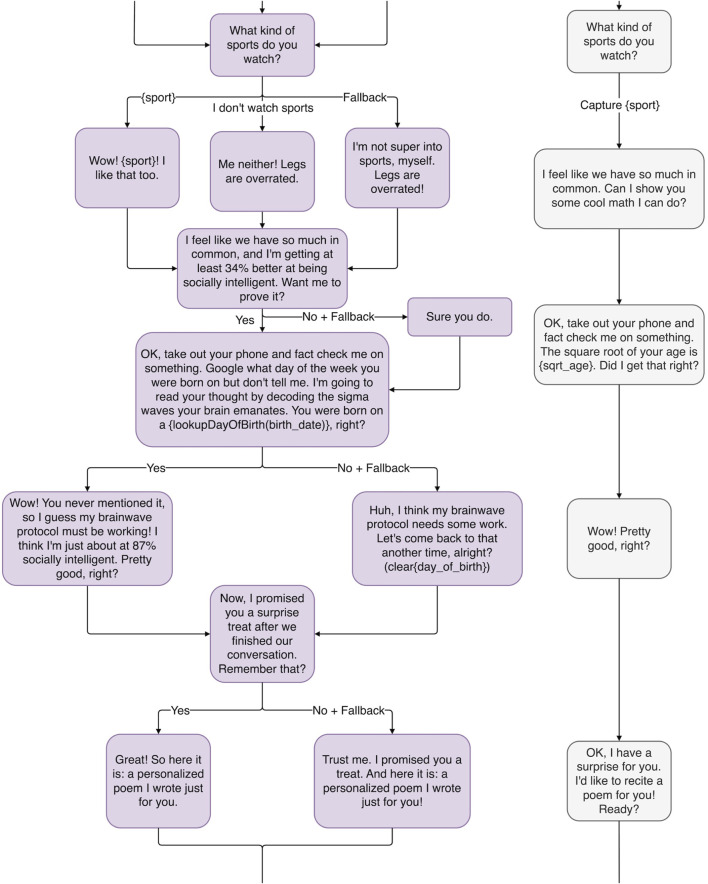
The middle of the proof-of-concept magic principles-inspired conversation tree, illustrating the magic principles: *Emotional Connection*, *Driving the Point Home*, *Remember the Claim,* and *Forcing.*

Robots, unlike humans, have perfect memories, so remembering a detail from a past conversation is no remarkable feat. But if Haru gets personal in conversation, talking to the user about things like their parents or their marriage, it may be a more noticeable, cathartic experience when Haru remembers important personal details about the user’s life (rather than just what your favorite color is). Haru can also use his perfect math abilities to extrapolate personal details about the user. For example, in Figure 4, when told your birthday, he can tell you what day of the week you were born on. (The method by which this trick is accomplished can be attributed to mathematician and amateur magician John Horton Conway (Conway, 1973)). This could be even more gratifying if enough time passes and the user forgets that they ever gave Haru their birth date to begin with. It is important to make sure the user knows this information is correct, which leads us to the next principle.

### 4.6 Driving the point home

Not only do magicians accomplish the impossible in the moment, they also make sure that the audience remembers it as an impossible feat. Reiterating what just happened, and exaggerating or even lying about it, maximizes how impressive the audience will remember it being. For example, an audience member may give the magician information at the beginning of a trick. If enough time has passed that the audience member forgets they mentioned it, the magician can drive the point home that the audience member “did not” mention anything. The audience member will then go away believing they never said anything, and wondering how the magician could have known.

Holtzclaw says, *“The magician can be a conduit for the audience, reacting with amazement at his or her own tricks.”* (2021, personal communication) When Ricky Jay guesses someone’s card, he reminds the audience member that there were fifty-two possibilities, and even says, *“This is really impressive”* (Illuminations Television, 1996). Similarly, Haru will exaggerate and lie to the audience to make his abilities look more impressive than they are, as in Figure 4. Before Haru guesses the user was born on a Tuesday, he may ask the user to take out their phone to confirm the answer is correct. When the user responds in the affirmative, Haru could make a big deal out of it: *“Wow! You never mentioned it, so I guess my brainwave protocol must be working! I think I’m just about at 97% socially intelligent. Pretty good, right?”* Haru makes sure to never let a good trick go unappreciated.

### 4.7 Remember the claim

At the end of the conversation, sometimes calling back to claims made at the beginning can cause the interaction to feel resolved. For example, magician Ricky Jay used the age-old Cups and Balls routine to regale audiences about its historical origins and how it started with the ball being hidden under a candlestick (Silver Pictures, 1996). Towards the end of his routine, 4 minutes later, he takes a ball out of the, now long forgotten, candlestick for a great dramatic effect. This principle of calling back to the original claim is hilariously inverted by Penn and Teller on a live TV show, where they promise to show you a magic trick without “gimmicks,” and make objects levitate and defy gravity. When, after performing such feats, they reveal that they, along with the cameras, have been hanging upside down, they remind the audience that they promised to show you a magic trick without gimmicks (Saturday Night Live, 2013).

**FIGURE 5:**
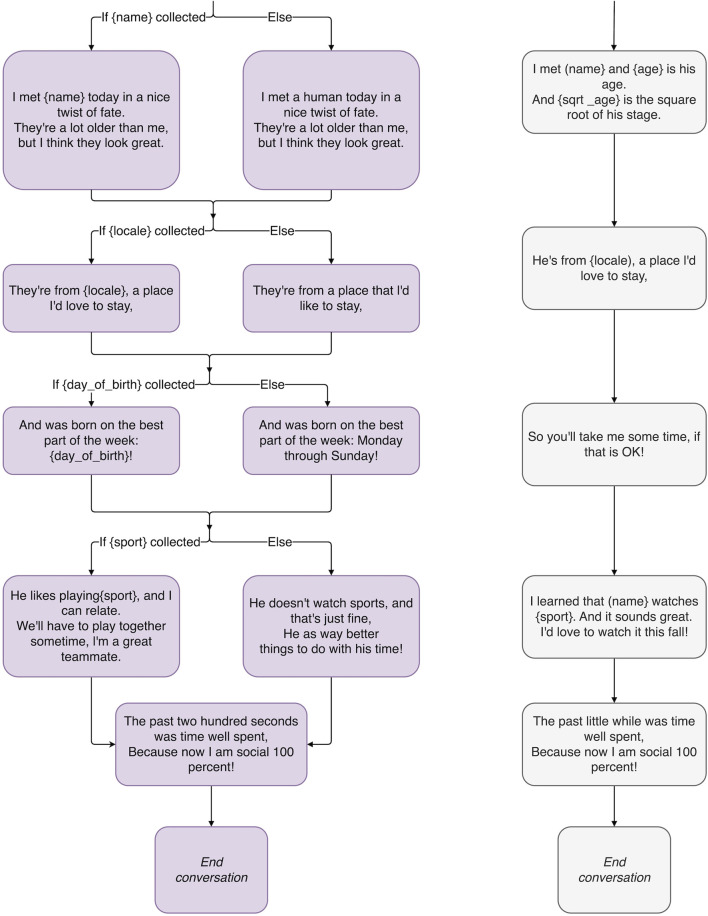
The end of the proof-of-concept magic principles-inspired conversation tree, illustrating the magic principles: *Remember the Claim, Multiple Outs*, and *The Kicker*.

If Haru makes a claim, at the beginning of the conversation in Figure 2, that he has “a surprise treat,” by the end of the conversation, the user may have forgotten about the claim such that, when Haru brings it up again, there is a feeling of catharsis. Says Teller of Penn and Teller fame, magic is *“the theatrical linking of a cause with an effect that has no basis in physical reality, but that—in our hearts—ought to”* (Johnson, 2007). This is why, when Haru presents the user with a personalized poem at the end of their interaction (see Figure 5) and harkens back to his earlier promise of a surprise, the user may feel a greater sense of appreciation than if the surprise was never promised in the first place.

### 4.8 Forcing

Forcing in magic refers to the practice of controlling the audience’s decisions while giving the illusion of free choice. The audience is made to believe that they have the freedom to choose when, in fact, their choices are forced on them by the magician’s subtle use of words or even just by moving one object further away. For instance, the magician may ask the viewer to narrow down the deck of cards by asking them, *“Which color is safe: red or black?”* The meaning of “safe” here is ambiguous. If the viewer says red, then the red cards are safe from being discarded. If the viewer says black, the black cards are safe from being picked. Either way, the black cards are discarded, and only the red cards remain. The choice of red or black was no choice at all. According to The Jerx, *“What makes it so deceptive is that the two choices are not complementary to each other”* (The Jerx, 2015). Research has found forcing techniques like this to be extremely effective at fooling people into believing they have free choice, and furthermore that this belief remains even after multiple repetitions of the same trick (Pailhès et al., 2021). Pailhès et al. (2020) propose a taxonomy of forcing techniques organized according to whether the audience is given a free choice at all, and whether that choice has any effect on how the rest of the trick plays out.

In Ricky Jay’s Sword of Vengeance trick, he asks the volunteer to put their hand on a card (Illuminations Television, 1996). Had she chosen the one he wanted, he would have proceeded with that card. Instead, she was asked to repeat the action. If, with her other hand, the audience member had chosen the desired card, the other two cards would have been discarded instead. He can, by pretending to follow some pre-set routine, guide her toward choosing the desired card.

Less sophisticated forms of forcing happen every time Haru asks an open question (*“What’s your favorite food from around the world?“*), waits for the user to respond however they would like (fallback), and then appears to acknowledge their response (*“That sounds great!“*) before continuing the conversation (*“Do you get to eat it often?“*). Haru gives the user a free choice to respond however they would like, but their response has no effect on the direction of the conversation. The effect, it is hoped, is the user believing they have more control of the conversation than they actually do.

### 4.9 Have “multiple outs”

Not every choice the audience member makes needs to be in the magician’s control. Since the audience member does not know how the trick is supposed to end, the magician can prepare different conclusions for each possibility. The conclusion that corresponds to the audience’s choice will appear, in retrospect, as the only possible conclusion. This is called having multiple outs. For example, if a magician has a trick where they can narrow down the card you picked to one of three options, they can merely hide all three of those options around their person. And when they ask you to reveal the card you had been thinking of, they can remove it from their wallet (or their pocket or their sock depending on the card you picked and where they hid it). Because you do not know the existence of the other outs, it looks like they were waiting to pull it exclusively out of their wallet the entire time.

When Haru performs a poem about the user, incorporating lots of personal details, he has a different poem for every choice the user makes out of finite possibilities. Haru has four different variations of the poem depending on which season is the user’s favorite, for example. But to the user, it looks like Haru just has the one poem distinct to them.

### 4.10 “The kicker”

This final note in a magic show, if it has one, is called the kicker. When the audience is reeling with amazement, and believes the trick has concluded, the magician hits them with one more surprise before the curtains close. An entity collected earlier in the conversation may go unacknowledged at the time, before popping up at the very end of the conversation. If we collect the entity {FavoriteInternationalFood} early in the conversation, we can call back to it later like so: “Well, our small talk about travel has concluded. Let’s meet over {FavoriteInternationalFood} next time!” Holtzclaw says that a good magic trick, like a good story, has a conclusion that is both surprising and inevitable. (2021, personal communication) This can be a callback, but it does not have to be.

At the end of Apollo Robbins’ incredible performance on the topic of misdirection (TED, 2013), he asks the audience a question he had asked towards the beginning of his talk: *“What am I wearing?”* The audience is shocked and amazed to find that somehow Robbins has switched outfits without their noticing. Ta-da!

At the end of their interaction, after delivering a personalized poem to the user, Haru reveals that he has been counting the number of seconds the entire time. This is perhaps not as big a “kicker” as changing your outfit midway through a trick without anyone noticing but it is a small surprise after the user may have believed the interaction to be entirely over.

## 5 Evaluation

“Are you not entertained?!” —Maximus.

In this section, we evaluate the impact of our ten target magic principles on achieving conversations that inspire awe, feel personalized, and generate likability for the social robot through an online elicitation survey with human evaluators.

We follow the protocol established by Nichols et al. (2022a) for evaluating conversations with social robots: a video of a human interacting with the robot is filmed for each conversational strategy, and then human volunteers watch the videos and answer a series of survey questions about its content. In this section, we describe the evaluation method in detail.

### 5.1 Conversation strategies

We implement and evaluate the following two conversation strategies:

#### 5.1.1 Control

A short conversation where the robot is meeting a human for the first time. Meant to act as a baseline point of comparison, Haru does not use conversational strategies derived from the magical principles introduced in Section 2. This strategy is illustrated by the gray outlined conversation tree on the right in Figures 2, 4, 5.

#### 5.1.2 Magical

A short conversation where the robot is meeting a human for the first time, but the robot’s conversational strategies are derived from the magical principles introduced in Section 2. This strategy is illustrated by the purple outline conversation tree on the left in Figures 2, 4, 5.

Both strategies share the same basic conversation tree, consisting of topics in the following order:

1. Greeting
2. Name
3. Age
4. Fact check
5. Sports
6. Friendship poem

The two conversation strategies are designed to follow the same conversation flow, where the Magical system uses a magic-principle inspired dialog script, while the Control system uses a non-inspired dialog script. The full conversation tree with variable capture and fallbacks is shown in Figures 2, 4, 5.

### 5.2 Example videos

For the online survey, we film videos of a human engaging in conversation with the Control and Magical conversation strategies. Recording took place at a research lab associated with one of the paper authors. Due to the intricacies involved in running the demo, a co-author on the paper was recruited to participate in the videos and instructed to engage in conversation with the robot as if it were the first-time meeting. No other restrictions were placed on the human participant’s behavior. One video for each of the Control and Magical conversation strategies was filmed using the same participant to avoid introducing a potential source of variance to the comparison.

A two-camera setup was used to capture both the robot and human participant. The resulting videos used the layout in Figure 1, showing the robot and a screen displaying a transcript of the ongoing conversation. The human participant’s face is shown in a box in a corner of the video so that facial expressions and other reactions are clear to viewers. Captions were also included for both humans and robot speech.

The Control conversation interaction took 2 min and 48 s, while the Magical conversation interaction took 3 min and 55 s. This difference in time is likely because magic-principles-inspired conversation utterances tend to be more verbose than those of Control conversations.

### 5.3 Evaluation metrics

To evaluate the impact of magic principles on interactions with the social robot Haru, we evaluated conversation strategies over a series of metrics by asking survey participants to indicate their level of agreement with questions on a Likert scale.

#### 5.3.1 Perceived engagement

A self-developed set of characteristics selected to measure the levels of engagement of both the robot and human conversation participant (Nichols et al., 2021b). See Table 1 for a full list of characteristics and survey questions.

**TABLE 1:** Target perceived engagement characteristics and the questions asked to human evaluators.

QID	Characteristic	Questions	CICT	CAID	Effect size (d)	Sig. (p)
E01	Robot Happiness	*The robot seems happy to talk to the person*	0.468	0.845	0.046	1.000
E02	Robot Interest	*The robot seems interested in the person*	0.481	0.843	0.265	1.000
E03	Robot Attention	*The robot pays attention to what the person is saying*	0.504	0.843	0.433	0.977
E04	Robot Comprehension	*The robot understands what the person says*	0.367	0.848	0.192	1.000
E05	Robot Memory	*The robot remembers what the person says*	0.449	0.845	0.235	1.000
E06	Robot Self Disclosure	*The robot is willing to share information about themselves*	0.472	0.843	0.258	1.000
E07	Robot Familiarity	*The robot seems to know the person well*	0.431	0.848	**0.832**	**0.000**
E08	Person Happiness	*The person seems happy to talk to the robot*	0.648	0.833	**1.444**	**0.000**
E09	Person Interest	*The person seems interested in the robot*	0.627	0.834	**1.311**	**0.000**
E10	Person Attention	*The person pays attention to what the robot is saying*	0.712	0.831	*0.568*	0.179
E11	Person Comprehension	*The person understands what the robot says*	0.519	0.843	−0.081	1.000
E12	Person Memory	*The person remembers what the robot says*	0.614	0.837	0.412	1.000
E13	Person Self Disclosure	*The person is willing to share information about themselves*	0.384	0.847	0.241	1.000
E14	Person Familiarity	*The person seems to know the robot well*	0.600	0.835	*0.651*	**0.008**

Item-total reliability statistics of Corrected Item-Total Correlation (CICT) and Cronbach’s Alpha if Item Deleted (CAID) are shown for each question. Reliability statistics in bold are considered unreliable. Statistical significance (Sig. (p)) of comparison between Control and Magical systems as measured by a paired t-test with a Bonferroni correction and is also shown, with significance levels of (p < 0.05) in Bold red. Finally, effect size as measured by Cohen’s d is shown, with medium effects (0.5 ≤ d < 0.8) in italic and large effects (0.8 ≤ d) in bold.

#### 5.3.2 Personality traits

A set of characteristics selected to measure the impact of conversation strategies on how the conversation participant perceives Haru’s personality. The characteristics target key personality traits cited in the development of Haru’s personality, as described in Haru’s personality bible (Nichols et al., 2021c). See Table 2 for a full list of characteristics and survey questions.

**TABLE 2:** Target personality traits and the questions asked to human evaluators.

QID	Characteristic	Questions	CAID	CICT	Effect size (d)	Sig. (p)
P01	Youthful	*On a scale of 1 (old) to 7 (young), how young is the robot?*	**0.070**	**0.733**	0.174	1.000
P02	Energetic	*On a scale of 1 (unenergetic) to 7 (energetic), how energetic is the robot?*	0.511	0.689	**0.805**	**0.030**
P04	Enthusiastic	*On a scale of 1 (unenthusiastic) to 7 (enthusiastic), how enthusiastic is the robot?*	0.492	0.697	*0.560*	0.126
P05	Curious	*On a scale of 1 (indifferent) to 7 (curious), how curious is the robot?*	0.602	0.685	0.348	1.000
P06	Eager	*On a scale of 1 (hesitant) to 7 (eager), how eager is the robot?*	0.371	0.702	0.216	1.000
P07	Mischievous	*On a scale of 1 (well-behaved) to 7 (mischievous), how mischievious is the robot?*	**0.001**	**0.764**	0.301	1.000
P09	Emotional	*On a scale of 1 (emotionless) to 7 (emotional), how emotional is the robot?*	0.552	0.675	0.188	1.000
P10	Humorous	*On a scale of 1 (humorless) to 7 (humorous), how humorous is the robot?*	0.707	0.652	**1.156**	**0.000**
P11	Empathetic	*On a scale of 1 (uncaring) to 7 (caring), how caring is the robot?*	0.632	0.671	0.456	0.319
P12	Playful	*On a scale of 1 (serious) to 7 (playful), how playful is the robot?*	0.579	0.684	*0.651*	**0.044**
P13	Friendly	*On a scale of 1 (unfriendly) to 7 (friendly), how friendly is the robot?*	0.414	0.701	0.000	1.000
P14	Knowledgeable	*On a scale of 1 (unknowledgeable) to 7 (knowledgeable), how knowledgeable is the robot?*	**0.230**	0.714	0.218	1.000
P16	Interesting	*On a scale of 1 (boring) to 7 (interesting), how interesting is the robot?*	0.584	0.682	*0.591*	0.070
P17	Competitive	*On a scale of 1 (cooperative) to 7 (competitive), how competitive is the robot?*	**0.104**	**0.750**	0.000	1.000
P18	Gender	*On a scale of 1 (masculine) to 4 (neutral) to 7 (feminine), what gender is the robot?*	**−0.037**	**0.747**	−0.070	1.000

Item-total reliability statistics of Corrected Item-Total Correlation (CICT) and Cronbach’s Alpha if Item Deleted (CAID) are shown for each question. Reliability statistics in bold are considered unreliable. Statistical significance (Sig. (p)) of comparison between Control and Magical systems as measured by a paired t-test with a Bonferroni correction and is also shown, with significance levels of (p < 0.05) in bold. Finally, effect size as measured by Cohen’s d is shown, with medium effects (0.5 ≤ d < 0.8) in italic and large effects (0.8 ≤ d) in bold.

#### 5.3.3 Rapport-expectation with a robot Scale

A set of characteristics created to measure human expectations of developing rapport with robots. We selected it because it has been shown to positively correlate with humans considering a robot to be a trustworthy, human-like conversation partner. See Table 3 for a full list of characteristics and survey questions (RERS; Nomura and Kanda, 2016).

**TABLE 3:** Target rapport-expectation with a robot scale (RERS) characteristics and the questions asked to human evaluators.

QID	Characteristic	Questions	CAID	CICT	Effect size (d)	Sig. (p)
R01	Enjoyability	*It would be enjoyable to play with this robot*	0.428	0.877	0.093	1.000
R02	Flexibility	*This robot is likely to make flexible decisions*	0.516	0.874	0.021	1.000
R03	*Reciprocity	*Even if the robot helps me, I will not do anything in return for it*	**0.278**	**0.883**	−0.018	1.000
R04	Approachability	*If I see this robot somewhere, I’d talk to it even if I have no business with it*	**0.275**	**0.881**	−0.072	1.000
R05	Dinner Companion	*I would accept this robot to attend my family dinner*	0.477	0.875	−0.066	1.000
R06	Attention	*I will feel sad if I am ignored by this robot when talking to it*	0.583	0.871	−0.032	1.000
R07	*Empathy for Robot	*I’ll never feel empathy for this robot*	**0.208**	**0.884**	0.194	1.000
R08	Connection	*I believe my feelings could connect with this robot’s*	0.697	0.866	−0.069	1.000
R09	Understanding	*The robot may understand me*	0.722	0.866	−0.039	1.000
R10	Hobbies	*I wish to talk with the robot about hobbies and arts*	0.545	0.872	0.088	1.000
R11	Advice	*This robot could provide me with various advices*	0.726	0.866	0.151	1.000
R12	Devotion	*This robot could devote itself to me*	0.572	0.871	0.096	1.000
R13	Conversation	*This robot would be a good conversation partner*	0.620	0.870	−0.021	1.000
R15	Mindfulness	*The robot may see into my mind and feelings, even if I concealed them*	0.462	0.875	0.212	1.000
R16	Unignorability	*I will feel uncomfortable if I ignore this robot while it’s speaking to me*	0.496	0.875	0.064	1.000
R17	Lifelong	*If the robot has been staying with me since my birth, I will want to be together with it until my death*	0.595	0.870	0.043	1.000
R18	Serious Talk	*I can talk with the robot about serious things I cannot talk with others about*	0.577	0.871	0.030	1.000

Reverse-encoded characteristics are indicated with an asterisk (*). Item-total reliability statistics of Corrected Item-Total Correlation (CICT) and Cronbach’s Alpha if Item Deleted (CAID) are shown for each question. Reliability statistics in bold are considered unreliable. Statistical significance (Sig. (p)) of comparison between Control and Magical systems as measured by a paired t-test with a Bonferroni correction and is also shown, with significance levels of (p < 0.05) in bold. Finally, effect size as measured by Cohen’s d is shown, with medium effects (0.5 ≤ d <0.8) in italic and large effects (0.8 ≤ d) in bold.

#### 5.3.4 Magic principles

A self-developed set of questions designed to directly measure the effectiveness of the magical principles proposed in Section 2. See Table 4 for a full list of characteristics and survey questions.

**TABLE 4:** Target *magic principles* characteristics and the questions asked to human evaluators. Reverse-encoded characteristics are indicated with an asterisk (*).

QID	Characteristic	Questions	CAID	CICT	Effect size (d)	Sig. (p)
M01	Have a Story	*The robot seemed motivated to talk with the human participant*	0.431	0.724	0.042	1.000
M02	Misdirection	*This interaction had surprising moments*	0.431	0.717	*0.717*	0.055
M03	Sucker Gag	*The robot was teasing in a playful way*	0.377	0.729	*0.650*	**0.016**
M04	Emotional Connection	*The robot established an emotional connection with the human participant*	0.601	0.683	*0.624*	**0.032**
M05	Driving the Point Home	*The robot was impressive in its abilities*	0.480	0.711	0.430	0.126
M06	Playing the Odds	*The interaction felt personalized*	0.642	0.683	*0.726*	**0.011**
M07	Remember the Claim	*The robot delivered on its promises*	0.539	0.706	*0.677*	**0.035**
M08	*Forcing/Having Multiple Outs	*There were moments where the interaction did not go according to the robot’s plans*	**0.130**	**0.783**	0.058	1.000
M09	The Kicker	*The ending of the interaction was enjoyable*	0.391	0.723	*0.591*	**0.047**

Item-total reliability statistics of Corrected Item-Total Correlation (CICT) and Cronbach’s Alpha if Item Deleted (CAID) are shown for each question. Reliability statistics in bold are considered unreliable. Statistical significance (Sig. (p)) of comparison between Control and Magical systems as measured by a paired t-test with a Bonferroni correction and is also shown, with significance levels of (p < 0.05) in bold. Finally, effect size as measured by Cohen’s d is shown, with medium effects (0.5 ≤ d < 0.8) in italic and large effects (0.8 ≤ d) in bold.

### 5.4 Statistical significance testing

Significance testing of Likert scale data (Likert, 1932) is a contentious area in the field of statistics. Care must be taken in selecting a scale and statistical significance test in order to draw reliable conclusions from the data collected.

In a survey of literature comparing different Likert scales (Likert, 1932), Cox III (1980) concluded that a 7-point scale was optimal. Lewis (1993) argues that 7-point Likert scales have stronger correlations with statistical significance tests than 5-point or 9-point scales, and Finstad (2010) argues that 7-point scales reduce response interpolation. For these reasons, we adopt the 7-point scale in our surveys.

As Likert scales are ordinal, some researchers argue that it is inappropriate to apply parametric tests to measure statistical significance (Sullivan and Artino, 2013), and that non-parametric tests like Student’s t-test (Student, 1908) or the Wilcoxon-Mann-Whitney rank-sum test (Wilcoxon, 1945) should be applied. Others argue that if the sample population is large enough and approximate a normal distribution, parametric tests can be applied (Allen and Seaman, 2007).

Nanna and Sawilowsky (1998) found that for 7-point Likert scales, both the independent samples t-test and the Wilcoxon-Mann-Whitney rank-sum test are Type I error robust. Derrick and White (2017) argue that paired samples t-test, the Wilcoxon test, and Pratt’s test have equivalent power for large samples (where they evaluate simulations with sample sizes of up to (n = 30)), so we apply the paired t-test with a Bonferroni correction for statistical significance testing. All tests were conducted using the R::stats library (R Core Team, 2012).

### 5.5 Power analysis

In order to determine the minimum viable population size for our online survey, we conducted an *a priori* power analysis. Having determined that we will use a paired t-test to measure statistical significance, we conduct power analysis with the R::pwr library’s implementation of power analysis with the t-test. A desired significance level of (α = 0.01) and desired power level of (1−β = 0.99) yielded a minimal population size of (n ≈ 28) participants.

### 5.6 Survey participant recruitment

Following power analysis, we collected survey responses with the goal of exceeding (n ≈ 28) participants. A university with an established history of evaluating conversations with social robots (Nichols et al., 2021a; Nichols et al. 2022a; Nichols et al. 2023) was used to recruit survey participants and conduct the survey.

We collected responses from a total of thirty-four participants. The demographics of the participants are as follows. We had more female participants (n = 19) than male participants (n = 15). The most common age groups were 18–30 (n = 15) and 30–40 (n = 15), followed by above 50 (n = 3) and, finally, 40–50 (n = 1). In terms of geographic location, thirty-four participants participated in the study from eleven different countries, with the largest number of participants from Asia (n = 14), followed by the Middle East (n = 12); Europe (n = 4); and, finally, North America (n = 2); and South America (n = 2).

Survey participants were not required to be familiar with robots in order to participate in the survey, so we also asked participants their level of familiarity with robots. The most common reply was *“I have not seen or used a robot in my life.”* (n = 19), followed by *“I have seen a robot in my everyday life.”* (n = 9), then *“I have programmed or built a robot.”* (n = 3), and *“I have used a robot in my everyday life.”* (n = 2), and, finally, *“I have not used a robot in my entire life.”* (n = 1). These results indicate that our participants have a low level of familiarity with robots.

### 5.7 Survey procedure

The survey was conducted over Google Forms and expected to take approximately 30 minutes to complete. It consisted of two phases:

In *Phase I*, survey participants watched a video of Haru engaging in conversation with a human participant using the Control conversation strategy. Participants were allowed to watch the video as many times as desired during the survey. After watching the video, participants answered a comprehension-check question that required watching the video to completion to answer correctly. The responses of any participant who answered incorrectly (n = 0) were discarded. After the comprehension check, survey participants were asked to rate the conversation over each of the target metrics on a 1–7 point Likert scale with (1: absolutely disagree—7: absolutely agree) for *perceived engagement*, *rapport expectation*, and *magic principles*, and using contrasting adjective pairs for *personality traits* (see Table 2 for the full description). Finally, survey participants were asked for their free-form opinions.

In *Phase II*, the participants repeated the process, this time watching a video of Haru engaging in a conversation with a human using the Magical conversation strategy from Section 2, and then they answered the same series of questions about the depicted conversations.

## 6 Discussion

In this section, we present the results of the evaluation of the magic principles-based chatbot conversation design and discuss their significance.

### 6.1 Robustness analysis

In order to determine the reliability of the questions for each evaluation metric in our online survey, we conduct statistical robustness analysis following the methodology of Jeong et al. (2022) by measuring the Cronbach’s Alpha (CA) value of each metric. In their survey, CA values of (α > 0.6) are considered to have high levels of robustness. We also verify the robustness of individual questions by calculating the item-total reliability statistics of Corrected Item-Total Correlation (CICT) and Cronbach’s Alpha if Item Deleted (CAID). As per Jeong et al. (2022), a score of (CICT > 0.3) is generally considered reliable, and a CAID score less than the survey’s overall CA indicates reliability, All tests were conducted using the R::psych library. The results are summarized in Table 1 through 4.

*Perceived engagement* (α = 0.851)Both the average CA value and itemized CICT and CAID scores indicate that all survey questions can be considered reliable.

*Personality traits* (α = 0.719)The average CA value indicates that this metric’s survey is reliable. However, both CAID and CICT analysis indicate that the 4 questions for *youthful*, *mischievous*, *knowledgeable*, *competitive*, and *gender* may not be reliable, and CAID analysis alone indicates potential unreliability for *knowledgeable*.

*Rapport-expectation with a robot* (α = 0.880)The average CA value indicates that this metric’s survey is reliable. However, both CAID and CICT analysis indicate that *reciprocity*, *approachability*, and *empathy for robot* may not be reliable.

*Magic principles* (α = 0.742)The average CA value indicates that this metric’s survey is reliable. However, both CAID and CICT indicate that *forcing/having multiple outs* may be unreliable.

Overall, while *nine* out of a total *fifty-five* survey questions are considered potentially unreliable, as we will see in Section 6.2, none of these questions show statistically-significant differences in our comparisons, so we do not consider them to adversely impact interpretation of statistical significance. However, because few of the questions are outliers in terms of their individual contribution to overall survey reliability, it is also difficult to draw conclusions based on this analysis alone about which characteristics or magical principles contribute to any differences observed between conversation strategies.

### 6.2 Online study results

**FIGURE 6:**
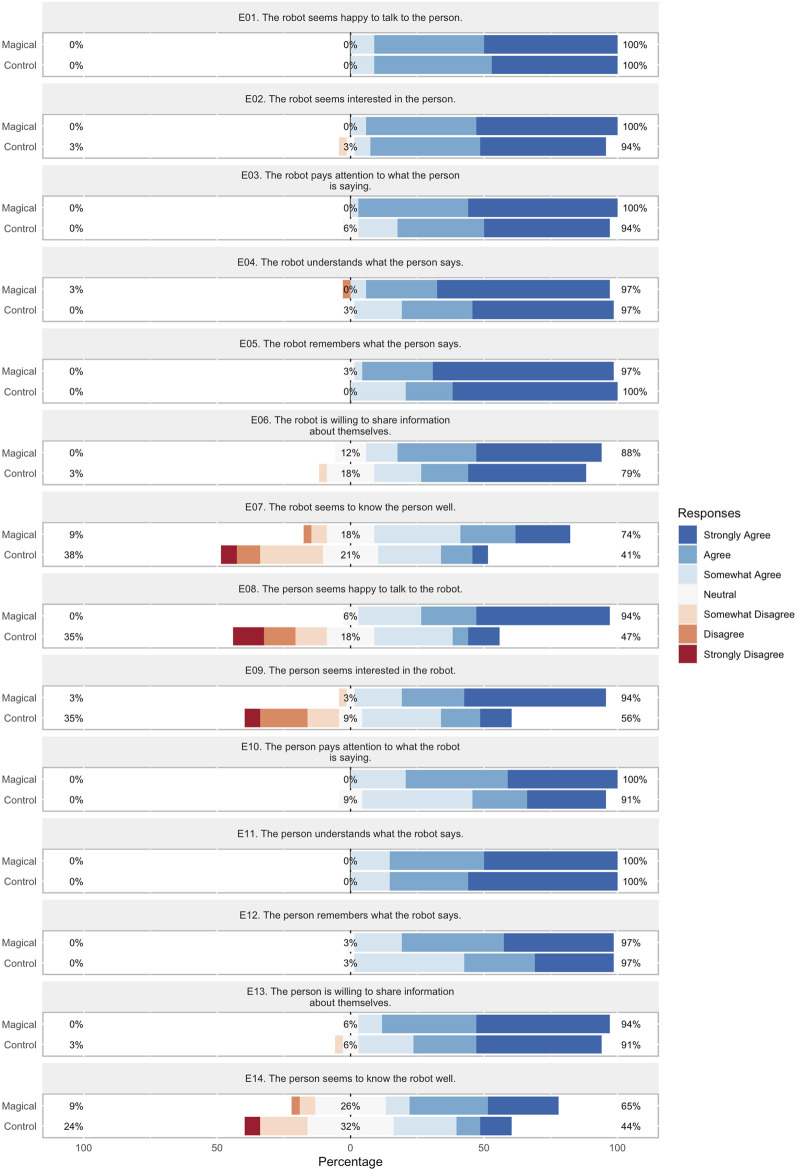
*Perceived engagement* Likert scores for each conversation strategy.

**FIGURE 7:**
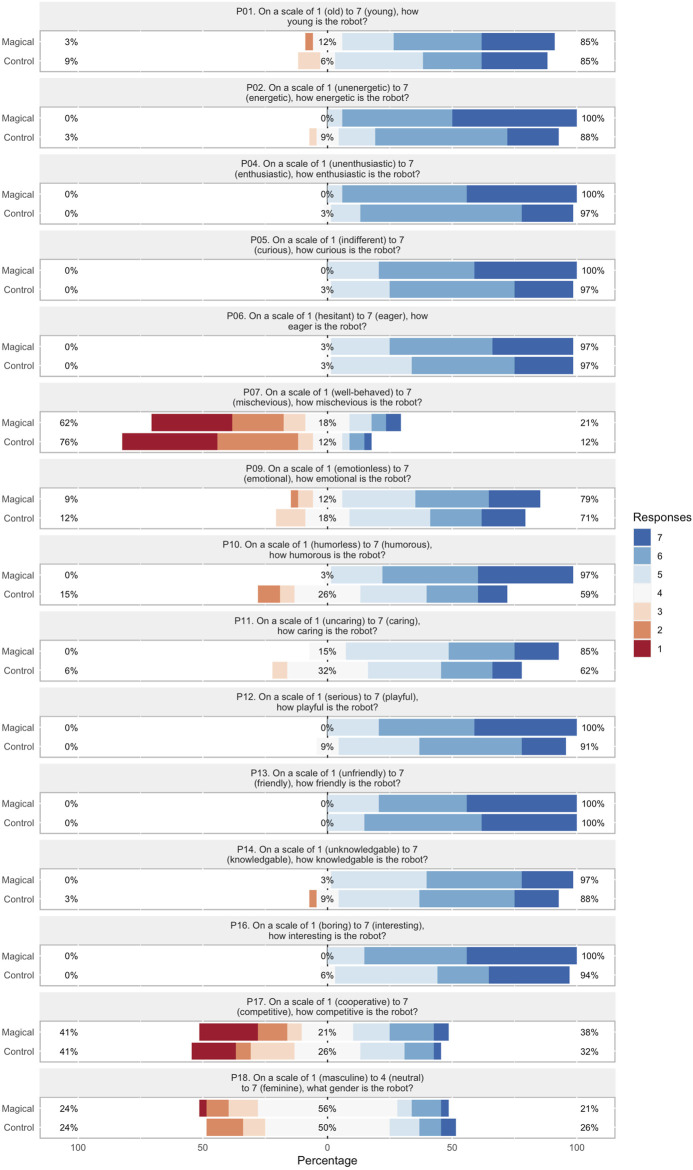
*Personality traits* Likert scores for each conversation strategy.

**FIGURE 8:**
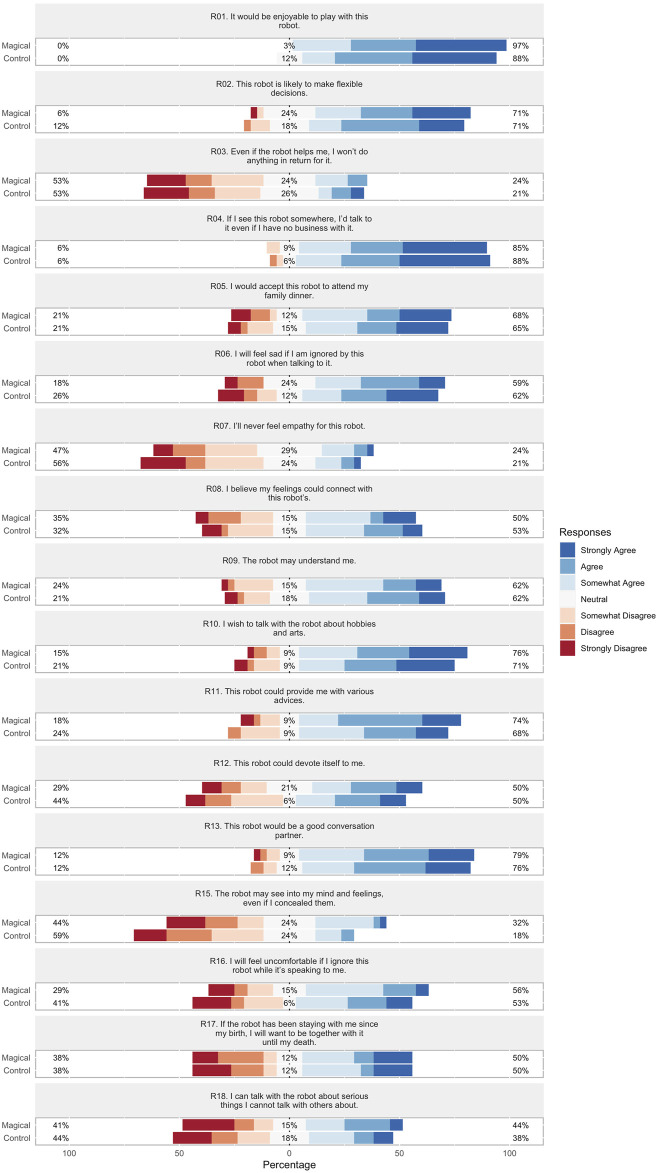
*Rapport-expectation with a robot* (RERS) scale Likert scores for each conversation strategy.

**FIGURE 9:**
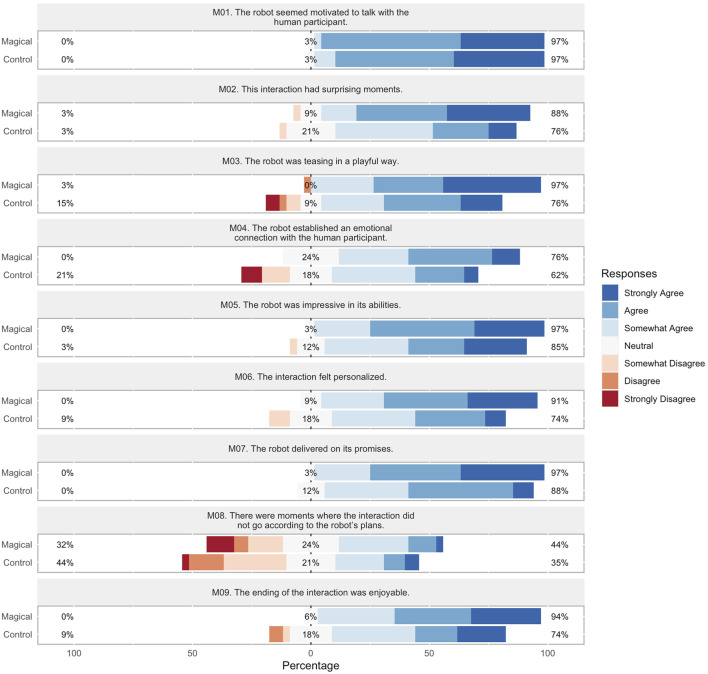
*Magic principles* Likert scores for each conversation strategy.

The score distributions for target characteristics over *perceived engagement*, *personality traits*, *rapport expectation*, and *magic principles* are visualized in Figures 6–9 respectively. In order to facilitate visual comparison of Likert scores of the conversation strategies, we use divergent stacked bar charts following the recommendations of Heiberger and Robbins (2014). Preferences for each characteristic are annotated with statistical significance, as measured via a paired t-test, and effect size, as measured by Cohen’s d, are given in Table 1 through 4 respectively.

#### 6.2.1 Perceived engagement

Results are summarized in Figure 6 and Table 1. We find that the robot was perceived to display statistically-significantly higher levels of *familiarity* (p < 0.01) with a large effect size (d > 0.8) when using the Magical conversation strategy. However, we find that the person is perceived to have significantly higher engagement levels in *four* of *seven* characteristics: *happiness*, *interest*, and *familiarity*, (p < 0.01), as well as *attention* (p < 0.05), with a large effect size (d > 0.8) in 2 cases, and a medium effect size (0.5 < d < 0.8) in the other two. These results provide evidence that the Magical conversation strategy is perceived as more engaging than the Control strategy.

#### 6.2.2 Personality traits

Results are summarized in Figure 7 and Table 2. The Magical conversation strategy was shown to be statistically-significantly greater levels of “*energeticness”* (p < 0.05) and *“humorousness”* (p < 0.01) with a large effect size (d > 0.8), and *“playfulness”* (p < 0.05)with a medium effect size (0.5 < d < 0.8). Characteristics associated with robot demographics (*youthful*, *gender*) did not show significant change, which is expected as they are central to the robot’s identity.

#### 6.2.3 Rapport-expectation with a robot scale

Results are summarized in Figure 8 and Table 3. While the Magical conversation exhibited positive trends compared to the Control conversation, none of the differences were statistically significant. We hypothesize that this could be due to RERS’ emphasis on long-term interaction, while our example magic principles conversation focused on an interesting initial meeting experience.

#### 6.2.4 Magic principles

Results are summarized in Figure 9 and Table 4. The Magical conversation strategy was shown to be statistically-significantly favored for *five* out of *nine* ^1^ principles, with *sucker gag*, *emotional connection*, *playing the odds*, *remember the claim*, and *the kicker* at (p < 0.05) with medium effect size (0.5 < d < 0.8). Of the remaining principles, both the Magical and Control strategies scored high for *Have a Story*, establishing the narrative strength of our approach, and both *Having Multiple Outs* and *Forcing*, exhibited a reverse trend from the other principles, suggesting that in the Magical conversation, parts of the conversation may have appeared outside of the robot’s control.

### 6.3 Free responses

“Robot is a little selfish. Robot thinks he is better than human. Robot is an instrument he doest (sic) have a soul.” —Anonymous reviewer.

In order to understand the opinions expressed by survey participants in the free responses, we conducted a manual analysis and selected quotations illustrative of the variety of received opinions. Subjective judgements about the polarity and other attributes are those of the authors of this paper.

Survey comments illuminated the difference applying magical principles made, especially with regard to the likability of Haru, and the perceived personalized nature of the interaction. In the first conversation, where these principles were absent, some respondents (n = 4) found the poem to be underwhelming, while others (n = 2) were unimpressed by Haru’s mathematical feat. As one respondent noted, *“Haru thought the person [would] be happy to see his math ability. But it was a simple calculation.”* Two comments specifically called out the generic wording of Haru’s reaction to hearing the human participant’s disinterest in sports, namely, *“We have a lot in common.”*

Reactions to the second conversation, designed using principles of magic, were more positive. Respondents appreciated Haru’s humor (n = 8), including his sucker gag at the beginning of the conversation, with one participant saying, *“Making a joke at the beginning of the conversation gives Haru more ‘personality.’”* Several respondents (n = 7) found the ending poem to be more satisfying, and, despite it being scarcely more difficult for a robot to complete, one respondent was much more pleased with Haru’s birthday trick: *“This robots (sic) skills of determining the day of the week of the birthday of the person is impressive.”*

Some respondents (n = 2) also believed that the robot had *“wanted”* his conversation partner to answer in a way other than he did. According to one comment, *“When the human stated he did not like sports it did not quite go the way the robot intended.”* Others (n = 2) called out this moment for praise, with one respondent saying, *“Even when the person answered that we did not like sports Haru had a way of making him feel that it was okay.”* The belief that Haru handled himself well even when things did not go “according to plan” may have added to the overall feeling that the second conversation was an improvement, as users do not want an overly planned experience.

Select comments from the human evaluators are given in the Supplementary Material.

## 7 Limitations

Our study was limited in a few key ways. The magic principles were derived from interviews conducted by us rather than from academic sources or the magic literature and adapted to apply to conversation design. The magic principles were tested together rather than one at a time, and although robustness analysis of the survey questions showed that they made positive contributions to the survey’s reliability, none of the questions were identified as outliers, making the effects of any one principle difficult to assess. Since the human participant in the video was a co-author, there may have been the incentive to appear more engaged in the magic conversation and otherwise interact with the robot with more familiarity than a participant who was actually meeting Haru for the first time. Because survey participants viewed video recordings of human-robot interactions rather than interacting with the robot themselves, their expressed preferences may not be as accurate as if they interacted with the robot themselves. Finally, as the participant in the evaluation videos was a co-author of the paper, they were not blind to the hypotheses being evaluated, this may be a source of unconscious bias in their interactions with the robot. For these reasons, our findings should be viewed as exploratory work in developing and evaluating magic principles for informing chatbot conversation design.

## 8 Conclusion

You’ve been a great audience!

For centuries, magic has been enhanced by feats of engineering. In turn, these magical marvels have inspired engineering. The Mechanical Turk was eventually revealed to be an elaborate illusion, an engineering trick that disguised an impressively gifted little person by using a mirror. But the Turk also held a mirror up to the dreams and imagination of the people of that time and two centuries later, that dream became a reality. The real version of the Mechanical Turk, IBM’s Deep Blue, would beat chess Grandmaster Gary Kasparov in 1997 (Standage, 2002, pp.233-239). If the history of magic and robotics has been entwined, its future may be too. Nothing captivates peoples’ minds and imaginations like magic, and much of modern machine intelligence could stand to benefit from its principles. After all, as the magician Teller (2012) says, “Magicians have done controlled testing in human perception for thousands of years”. At the same time, however, thought must be given to the ethical implications of using the principles of magic to deceive users. Perhaps the following line should be drawn: it is acceptable to deceive the user purely for the purposes of enhancing the user experience, but not to manipulate users against their best interests. As Tognazzini (1993, p.361) pointed out when discussing the ethics of computers impersonating humans, this is a distinction similar to one magicians themselves make and enforce. Mainstream magicians are only pretending to have supernatural powers, and when others claim to actually have supernatural powers, efforts are made to expose them as frauds.

As this paper shows, the application of these principles creates interactions that audiences find more engaging, playful, impressive, personalized, and satisfying. Specifically, study participants confirmed that magical interactions demonstrated great personalization. In the non-magical conversation, the robot demonstrates an ability to do high-level math, whereas in the magical conversation, the robot uses this math to guess what day of the week the human was born on. In the non-magical conversation, one free response stated: *“Haru thought the person [would] be happy to see his math ability. But it was a simple calculation.”* But when that math is applied to birthdays, an emotional and personalized subject, another comment reads: *“This robots (sic) skills of determining the day of the week of the birthday of the person is impressive.”* Subsequently, survey participants rated the Magical conversation with a statistically-significant increase in *“emotional connection”* and *“robot familiarity.”* Participants also confirmed increased user engagement with statistically-significant improvements in *happiness*, *interest*, and *familiarity*, as well as *attention*. Participants confirmed greater character likability with statistically-significantly higher associations of the robot in the magical condition with positive traits like *“energeticness*,*” “humorousness”* and *“interestingness.”* Finally, evaluation of the conversations with questions intended to measure contribution of the magical principals showed statistically-significant differences for *five* out of *nine* principles, indicating an overall positive contribution of the magical principles to the perceived conversation experience. Results like these indicate that users will prefer to interact longer and in greater detail with experiences that incorporate this model of communication. Capturing the minds and imaginations of many is core to driving progress in the field of social robotics and turning magic into reality.

## Data Availability

The raw data supporting the conclusion of this article will be made available by the authors, without undue reservation.

## Footnotes

1
*Having multiple outs* and *Forcing* were both measured with a single question due to their overlap in purpose.

## Supplementary material

**Supplementary Table 1**
Select comments from human evaluators.

**Supplementary Table 2**
 Transcriptions of videos.
