# Supplementary material for: Would you be impressed: applying principles of magic to chatbot conversations
Source: Front Robot AI. 2024 Apr 24;11:1256937. doi: 10.3389/frobt.2024.1256937 (PMC11076854; doi:10.3389/frobt.2024.1256937)
Supplement: Supplementary file 1 [file Table1.DOCX]

Supplementary Material

| **Most people thought Haru dominated the first conversation, and that Eric did not appear interested. (24 comments)** |
| --- |
| *“The conversation was ok. It wasn't that exciting but it wasn't bad. The robot should let the person talk more.”* |
| *“Haru looks really happy to know the person and talk with him. But the person looks not much interested to talk with Haru. He gives short answers.”* |
|  |
| **Some people could tell that Haru’s response when Eric said he didn’t like sports wasn’t personalized in the first conversation. (2 comments)** |
| *“I felt like the answer 'Wow we have alot in common' was generic”* |
| *“I thought the robot understood everything except when Eric said that he wasnt a fan of sports and Haru said we have alot in common after that.”* |
|  |
| **Some people were not impressed with the square root calculation in the first conversation. (2 comments)** |
| *“Haru thought the person will be happy to see his math ability. But it was a simple calculation. Then he tried to please eric by sing a poem. It also should be improved.”* |
| *“They are sharing only the basic information like name, where from. And I do not see any interest in knowing the square root of my age.”* |
|  |
| **Some people were not impressed by the poem in the first conversation. (4 comments)** |
| *“Ending could have been better. Felt a bit detached.”* |
| *“The song that the robot sang was not so interesting and the way he paused after every sentence made me a little boring”* |
|  |
| **Most people thought Eric enjoyed the second conversation much more. (28 comments)** |
| *“This conversation is more interactive between Robot and human.”* |
| *“Both human and robot seems interesting to talk to each other.”* |
| *“Erik was more active and I liked it”* |
|  |
| **A lot of people liked the sucker gag that opened the second conversation, and Haru’s jokes in general (8 comments)** |
| *“Making a joke at the beginning of the conversation gives Haru more 'personality.’”* |
| *“The person is much more interested to talk with Haru. And Haru is making practical jokes and they are very natural.”* |
|  |
| **Haru’s goal of becoming social intelligent (Have a Story)** |
| *“Haru seemed to achieve its goal--becoming more socially intelligent--in this conversation.”* |
|  |
| **The promise of a surprise (Remember the Claim)** |
| *“I also liked Haru's promise of a treat at the end of the chat. That intention to draw attention made the video more enjoyable to watch.”* |
|  |
| **The more sophisticated reaction to Eric saying he’s from the US (Playing the Odds)** |
| *“The surprising moments to me were 1) when Haru mentioned 'Liberty, Eagles' when Eric said he's from the US;”* |
|  |
| **The improved calendar trick (Establishing an Emotional Connection)** |
| *“This robots skills of determining the day of the week of the birthday of the person is impressive.”* |
|  |
| **The improved poem (Have “Multiple Outs”).** |
| *“I feel the poem is well advance and more creative compare to the first session conversation.”* |
|  |
| **Other interesting comments:** |
| Someone suggested something after watching the **Control** conversation video that was implemented in the **Magical** conversation video*: “It will be interesting if this robot knows some information about the countries. when the person says from where he is, then adding information abt that place by robot will be interesting for the person.”* |
| Someone thought Haru had to vamp in the **Magical** conversation video when Eric said he didn’t like sports*: “When the human stated he didn't like sports it didn't quite go the way the robot intended.”* |
| About the **Magical** conversation: “The interaction felt personalized but it seemed to be guided by the robot so I don't know if the robot is able to answer in any other way. The beginning of the talk had surprising moments. the robot seems to have sense of humor.” |
| About the **Magical** conversation*: “In my opinion, this robot has the potential to foster emotional connections with people. Its adorable and charming reactions contribute to this effect.”* |
| *“I didn't like those sounds haru made when he is happy.”* |

# Supplementary Table 1: Select comments from human evaluators.
