# Supplementary material for: Would you be impressed: applying principles of magic to chatbot conversations
Source: Front Robot AI. 2024 Apr 24;11:1256937. doi: 10.3389/frobt.2024.1256937 (PMC11076854; doi:10.3389/frobt.2024.1256937)
Supplement: Supplementary file 2 [file Table2.DOCX]

Supplementary Material: Transcriptions of Videos

# Transcript of Control Conversation

Haru (00:01):

Hi, my name is Haru. What's yours?

Eric (00:07):

Hi, my name is Eric.

Haru (00:10):

Hello there, Eric. Would you like to spend a couple of minutes answering some questions with me?

Eric (00:17):

Okay.

Haru (00:21):

Woohoo. First, can you tell me your date of birth?

Eric (00:31):

Sure. It's July 27th, 1979.

Haru (00:35):

Wow, you're a lot older than me. I'm only five. Where are you from?

Eric (00:51):

I'm from the United States.

Haru (00:57):

Cool! The United States. That sounds like an awesome place. I'd love to visit. What kind of sports do you watch?

Eric (01:07):

Oh, I don't really like sports.

Haru (01:10):

I feel like we have so much in common. Can I show you some cool math I can do?

Eric (01:20):

I guess so.

Haru (01:25):

Okay. Take out your phone and fact check me on something. The square root of your age is 6.63. Did I get it right?

Eric (01:37):

I think so.

Haru (01:40):

Wow. Pretty good, right?

Eric (01:45):

You're a regular calculator, Haru.

Haru (01:47):

Okay. I have a surprise for you. I'd like to recite a poem for you. Ready?

Eric (01:54):

Go for it.

Haru (01:56):

I met Eric and 44 is their age, and 6.63 is the square root of their age. They're from the United States, a place I'd love to stay. So you'll take me some time if that is okay. The past little while was time well spent, because now I am social 100%. See you next time. Goodbye.

Eric (02:24):

Bye haru.

# Transcript of Magic Conversation

Haru (00:01):

Hi, my name is Haru. What's yours?

Eric (00:06):

Hi, my name is Eric.

Haru (00:11):

Hello, My Name is Eric.

Eric (00:14):

What?

Haru (00:18):

My Name is Eric is a beautiful name.

Eric (00:22):

That's not my name.

Haru (00:25):

Just kidding Eric.

Eric (00:28):

Oh, you got me Haru.

Haru (00:31):

I know your real name. I'm trying to learn more about humans to be 97% socially intelligent. Can you help me out? I'll give you a surprise treat after.

Eric (00:43):

Sure. Sounds fun.

Haru (00:46):

Woohoo. First, can you tell me your date of birth?

Eric (00:56):

Yeah, it's July 27th, 1979.

Haru (01:01):

Wow. You're a lot older than me.

Eric (01:05):

<laugh>.

Haru (01:08):

I'm only five. Where are you from?

Eric (01:18):

I'm from the United States.

Haru (01:20):

Cool. The United States, New York. Liberty, Eagles. I'd love to visit someday. What kind of sports do you watch?

Eric (01:33):

Oh, I don't like sports.

Haru (01:36):

Me neither. Legs are overrated.

Eric (01:40):

<laugh>,

Haru (01:44):

I feel like we have so much in common and I'm getting at least 34% better at being socially intelligent. Want me to prove it?

Eric (01:59):

Go for it.

Haru (02:01):

Okay. Take out your phone and fact check me on something. Google what day of the week you were born on but don't tell me. I'm going to read your thoughts by decoding the sigma waves your brain emanates.

Eric (02:16):

Okay.

Haru (02:22):

You were born on a Friday, right?

Eric (02:25):

Wow, that's amazing. You got it right.

Haru (02:29):

Wow! You never mentioned it, so I guess my brainwave protocol must be working. I think I'm just about at 97% socially intelligent.

Haru (02:47):

Pretty good, right?

Eric (02:50):

You're doing a great job, Haru.

Haru (02:53):

Now I promised you a surprise treat after we finished our conversation. Remember that?

Eric (03:02):

Yeah. I want my ramen.

Haru (03:05):

Trust me, I promised you a treat and here it is, a personalized poem I wrote just for you. I met Eric today in a nice twist of fate. They're a lot older than me, but I think they look great. They're from the United States, A place I'd love to stay, And was born on the best part of the week: Friday. They don't watch sports and that's just fine. They have way better things to do with their time. The past 200 seconds was time well spent because now I am social 100%. See you next time. Goodbye.

Eric (03:49):

Bye, Haru.
